# The Great Saphenous Vein Proximal Part: Branches, Anatomical Variations, and Their Implications for Clinical Practice and Venous Reflux Surgery

**DOI:** 10.3390/jcdd11080242

**Published:** 2024-08-07

**Authors:** Krisztina Munteanu, Ovidiu Ghirlea, Daniel Breban-Schwarzkopf, Alexandra-Ioana Dănilă, Roxana-Georgeta Iacob, Ioan Adrian Petrache, Gabriel Veniamin Cozma, Anca Bordianu, Sorin Lucian Bolintineanu

**Affiliations:** 1 Department of Anatomy and Embryology, “Victor Babeş” University of Medicine and Pharmacy Timisoara, Eftimie Murgu Square No. 2, 300041 Timisoara, Romania; krisztina.munteanu@umft.ro (K.M.); daniel.breban-schwarzkopf@umft.ro (D.B.-S.); alexandra-danila@umft.ro (A.-I.D.); roxana.iacob@umft.ro (R.-G.I.); s.bolintineanu@umft.ro (S.L.B.); 2 Abdominal Surgery and Phlebology Research Center, “Victor Babeş” University of Medicine and Pharmacy Timisoara, 300041 Timisoara, Romania; 3 Department of Surgical Semiology, Faculty of Medicine, “Victor Babeş” University of Medicine and Pharmacy Timisoara, Eftimie Murgu Square No. 2, 300041 Timisoara, Romania; ioan.petrache@umft.ro (I.A.P.); gabriel.cozma@umft.ro (G.V.C.); 4 Thoracic Surgery Research Center, “Victor Babeş” University of Medicine and Pharmacy Timisoara, Eftimie Murgu Square No. 2, 300041 Timisoara, Romania; 5 Department of Plastic Surgery and Reconstructive Microsurgery, “Bagdasar-Arseni” Emergency Hospital Bucharest, “Carol Davila” University of Medicine and Pharmacy Bucharest, 020021 Bucharest, Romania; anca.bordianu@gmail.com

**Keywords:** lower limb vein anatomy, tributaries, venous aneurysm, anterior accessory great saphenous vein, venous reflux

## Abstract

The anatomical variations in the lower limb veins play a critical role in venous reflux surgeries. This study presents an analysis of the great saphenous vein (GSV) proximal part’s anatomical peculiarities, with 257 patients included, who were operated for venous reflux. This study highlighted a progressive increase in the GSV diameter in conjunction with the complexity of the anatomical variations, ranging from no tributaries to more than five tributaries, an anterior accessory GSV, or venous aneurysms. Statistical analysis evidenced this expansion to be significantly correlated with the variations. Additionally, the progression of the chronic venous disease (CVD) stages was notably more prevalent in the complex anatomical variations, suggesting a nuanced interplay between the GSV anatomy and CVD severity. Conclusively, our research articulates the paramount importance of recognizing GSV anatomical variations in optimizing surgical outcomes for CVD patients. These insights not only pave the way for enhanced diagnostic accuracy but also support the strategic framework within which surgical and interventional treatments are devised, advocating for personalized approaches to venous reflux surgery.

## 1. Introduction

The circulatory system can be divided based on blood vessel types. The venous system comprises blood vessels, which carry blood from tissue and organs, allowing a return to the heart [1]. In order to ensure blood return from the lower part of the body, the distal veins have a valve system that segments the blood column [2]. Venous valves play a crucial role in preventing pathologic reflux and maintaining a net vertically directed flow by preventing reflux. The valves also divide the hydrostatic column of blood into segments and prevent the full pressure of the fluid column from exerting force on the distal veins [3]. Valvular insufficiency leads to venous reflux in the lower extremities, which is a manifestation of a degenerative process in the venous wall [4] and supporting fascial structures, which progressively dilates over time after exposure to high physiological pressures [3]. With venous reflux progression, varicose dilatations of the veins occur.

Varicose dilatations of the veins are considered to occur in four anatomic regions extending in a descending fashion from the renal veins to the lower extremities [5]. The reflux occurring in these first three proximal topographical regions, namely the left renal vein, the gonadal and internal iliac veins and associated pelvic venous plexuses, and the pelvic-originating extrapelvic veins refluxing through the pelvic escape points to the genitalia and lower extremity veins, is related to pelvic venous disorders, which are rarely encountered compared with the fourth region venous reflux. Although often communicating with the pelvic-originating extrapelvic veins and refluxing through the pelvic escape points to the genitalia and lower extremity veins, the fourth area, consisting of the superficial and deep veins of the lower extremity and their tributaries, is much more commonly the site of venous valvular insufficiency [6]. According to the literature data, a much larger number of patients complain of lower limb venous reflux than patients suffering from the first three areas of venous reflux [5,6]. The presence of venous reflux at the level of the fourth topographic region leads to the onset of chronic venous disease (CVD), a prevalent condition worldwide, which has a significantly negative impact on the patient’s quality of life [7]. The incidence of varicose veins is approximately 2% per year, and the prevalence is higher in Western countries [8].

Venous reflux in the lower extremity is a common clinical problem, with objective clinical manifestations of CVD ranging from mild to severe, such as telangiectasia, oedema, lipodermatosclerosis, and, most notably, varicose veins, and the symptoms include (but are not limited to) leg pain, discomfort, heaviness, and swelling, itching, and burning paresthesias [3,9]. Without proper treatment, CVD progresses to skin changes, with venous leg ulcerations invariably occurring [10]. 

Patients initially seek treatment to relieve symptoms. It has been proven that several conservative measures like phlebotonic medication (pure diosmin being widely used) and compression stockings (the mainstay of conservative treatment) are effective in symptom relief and quality of life improvement [11,12]. However, curative treatment involves venous reflux closure. The modern surgical approach to varicose vein treatment is represented by venous reflux surgery. Whether dealing with a truncal or tributary reflux, it should be interrupted in order to prevent complications. The insufficient veins must be occluded or removed [13]. A significant percentage of patients with CVD require surgical vein ablation as a therapeutic method [14]. Stripping operations and the less invasive endovenous thermal ablation have shown comparable results in saphenous vein insufficiency treatment [15]. Different surgical and interventional methods are available, including conventional saphenectomy, cryostripping, endovenous laser or radiofrequency ablation, microwave or mechano-chemical ablation, VenaSeal (cyanoacrylate adhesive closure), foam sclerotherapy, and others. Although the landscape of chronic venous disease treatment has evolved, ushering in alternative methods that prioritize minimally invasive techniques, open surgical procedures are still the first treatment option in most of the low- and middle-income countries [16]. A basic understanding of the venous anatomy of venous reflux is essential for choosing the appropriate treatment strategy [3].

The venous anatomy of the lower extremity is substantially more variable and complicated than the corresponding arterial anatomy. Most anatomical variations are encountered in the proximal part of the great saphenous vein (GSV). The GSV is the longest vein in the human body, arises from the medial aspects of the dorsal pedal venous arch, and empties into the femoral vein just below the inguinal ligament, through the saphenous cross. The GSV is often accompanied by tributaries, and at times, the tributaries can be confused with the GSV or be mistaken for GSV duplication. Accessory saphenous veins, tributaries of the great saphenous vein cross (the most important and frequently encountered being a superficial circumflex iliac vein; epigastric vein; external pudendal veins; superficial dorsal veins of the scrotum; veins of the clitoris) must be identified and treated in case of venous reflux. However, the classic anatomy can only be noted in about 16–25% of the population [17]. 

This article aims to present a series of peculiar anatomical features of the great saphenous vein cross, which may be encountered during venous reflux surgery, as well as the challenges raised by those cases and how those should be approached.

## 2. Materials and Methods

### 2.1. Study Design and Patients

This research was an observational retrospective study, which assessed the anatomical peculiarities of the great saphenous vein proximal part (GSV cross and its collaterals). The clinical charts and the surgical records of 316 patients diagnosed with CVD, who were admitted to the Phlebology Department (1st Surgical Department, “Pius Brînzeu” Emergency County Hospital Timișoara, Romania) between January 2019 and December 2023, were analyzed (all the patients who presented with CVD during the defined time period were initially included).

The diagnosis was established in the clinical setting and was completed by ultrasound examination. All the patients were evaluated before surgery using the ‘pulse-wave’ Doppler exam, with the venous reflux at the GSV level noted. The evaluated patients subsequently underwent surgical treatment, with open procedures (conventional stripping, cryostripping, or phlebectomies) performed according to the therapeutic indication. 

### 2.2. Data Collection

The following data were collected: demographic data—gender, age, native environment (urban/rural); clinical data—body mass index (BMI) and CVD stage according to CEAP classification [18]; vein morphology and anatomical data (GSV diameter, GSV proximal part’s number of tributaries, different anatomical aspects compared with the classic anatomy). All the data were entered into a Microsoft Excel 2016 spreadsheet (Microsoft, Bellevue, WA, USA).

### 2.3. Enrolment Criteria

Considering the aim of our study, in order to achieve the necessary data, only the data of patients who underwent open corssectomy—incision, subcutaneous tissue dissection, GSV cross (saphenofemoral junction) dissection and exposure, tributaries dissection and ligation—were enrolled in the study. Thus, the patients in which only phlebectomies, laser endovenous therapy, or other conservative treatment methods were practiced were excluded. By applying these criteria, 257 patients were finally included in the study.

### 2.4. Statistical Analysis

Statistical analyses were completed using MedCalc^®^ Statistical Software version 20.118 (MedCalc Software Ltd., Ostend, Belgium; 2022). The measures of central tendency (mean), minimum, and maximum were determined, with data reported by rounding to two decimal places. The results were analyzed using the Mann–Whitney test and Chi-square test for nonparametric data. The normality of distribution was analyzed using the Kolmogorov–Smirnov test, and subsequently, comparisons of the parameter variations among the groups were performed using the ANOVA test. All *p*-values were two-sided, and a *p*-value < 0.05 was considered statistically significant.

## 3. Results

The gender distribution of the study population revealed a significant female predominance, with females constituting 71.9% (n = 185) of the patients, while males represented 28% (n = 72). Regarding the native environment, 55.2% (n = 142) of the patients were from urban areas, while 44.7% (n = 115) of the patients were from rural areas. The mean age of the participants was 53.2 years with a standard deviation (SD) of ±11.4 years, indicating a middle-aged cohort. The body mass index (BMI) of the study population averaged 26.9 kg/m^2^ (SD ± 4.7), suggesting a tendency towards overweight status among the participants.

Considering the clinical aspect (C) from the CEAP classification, the distribution of CVD stages among the patients was as follows: stage C2 was identified in 21% (n = 54) of the patients, indicating the presence of varicose veins without significant symptoms; stage C3, characterized by edema, was the most common, observed in 49% (n = 126) of the cases; stage C4, which involves skin and subcutaneous tissue changes, was noted in 24.9% (n = 64) of the participants; the C5 and C6 stages, indicative of healed and active venous ulcers, respectively, were less common, comprising 1.1% (n = 3) and 4.2% (n = 11) of the cases, respectively. These data are presented in Table 1.

Considering the anatomical variations in the GSV proximal part, the following cases were encountered: no tributaries in 0.3% (n = 1) of the patients; less than three tributaries in 25.2% (n = 65) of the patients; three to five tributaries in 63.8% (n = 164) of the patients; more than five tributaries in 10.5% (n = 27) of the patients. The superficial external pudendal vein and the superficial epigastric vein were the most frequently encountered branches, noted in 96.8% (n = 249) and 92.2% (n = 237) of cases, respectively. Other branches encountered were the superficial circumflex iliac vein in 51.75% of cases (n = 133), the deep external pudendal vein in 39.6% of cases (n = 102), the posteromedial thigh branch in 34.6% of cases (n = 89), the anterolateral thigh branch in 26.4% of cases (n = 68), and the medial accessory saphenous vein in 4.6% of cases (n = 12). Considering the topographic distribution of the branches, the medial ones—the superficial external pudendal vein (96.8%), the deep external pudendal vein (39.6%), the posteromedial thigh branch (34.6%), and the medial accessory saphenous vein (4.6%)—were more frequently encountered compared with the lateral branches—the superficial epigastric vein (92.2%), the superficial circumflex iliac vein (51.7%), and the anterolateral thigh branch (26.4%).

Additionally, an anterior accessory GSV (AAGSV) was also encountered in 12% (n = 31) of the patients, and an aneurysmally degenerated GSV (⌀ > 1.2 cm) was noted in 13.6% (n = 35) of the patients. In total, 20% (n = 7) of the cases diagnosed with GSV aneurysm were normoponderal patients, 51.4% (n = 18) were overweight patients, and 28.5% (n = 10) were obese patients. In addition, venous aneurysm was noted in 28.5% (n = 10) in the ‘<3 Tributaries’ group, in 60% (n = 21) in the ‘3–5 Tributaries’ group, and in 11.4% (n = 4) in the ‘>5 Tributaries’ group. Venous aneurysm seems to correlate with increased BMI and an increased number of tributaries, maybe as a result of the presence of tributaries’ reflux. All the above-described data are graphically presented in Table 2.

Overall, our analyses revealed significant correlations between anatomical variations of the GSV and a spectrum of demographic and clinical characteristics. These correlations are presented in Table 3.

In the realm of demographic and clinical characteristics, the study observed a gradient increase in both the mean Body Mass Index (BMI) and age across the anatomical variations, with the mean BMI elevating from 25 kg/m^2^ in the “No Tributaries” group to 30 kg/m^2^ in those identified with an “Anterior Accessory GSV”. This trend indicates a potential relationship between a higher BMI and more complex GSV variations. An elevated BMI could contribute to increased venous pressure, leading to endophlebohypertophy (marked cellular infiltration of the intimal layer, where the intima appears thicker than the media) and endophlebosclerosis and subsequent morphological changes in the GSV. The *p*-values for BMI, which ranged from 0.05 to 0.3, highlight meaningful differences across the groups, suggesting that BMI is an important factor in the development of these anatomical variations. The mean age of patients also showed significant variance across the anatomical variations. Patients in the “No Tributaries” group had a mean age of 55 years, whereas those in the “Anterior Accessory GSV” group had a mean age of 63 years. This trend suggests that age is a contributing factor to the complexity of GSV variations, possibly due to the cumulative effects of aging on venous structure and function. The *p*-values for age ranged from 0.04 to 0.09, indicating significant differences between the groups. The *p*-values for BMI and age were calculated using the ANOVA test, comparing the mean values across the different anatomical variation groups. This test helped determine whether there were statistically significant differences in BMI and age among the groups ‘No Tributaries’, ‘<3 Tributaries’, ‘3–5 Tributaries’, ‘>5 Tributaries’, ‘Aneurysmal Degenerate’, and ‘Anterior Accessory GSV’.

The GSV diameter was measured at the saphenofemoral junction level. While the diameter of a healthy GSV usually ranges from 2.5 to 6 mm, according to our findings, the insufficient GSV diameter ranged between 0.6 cm to 2.3 cm in the studied population, with an average of 0.93 ± 0.2 cm. Our analysis revealed a statistically significant increase in the diameter of the insufficient GSV, which correlates directly with the different anatomical variations. Notably, the diameter expanded from 0.9 cm in the singular instance of “no tributaries”, to an average of 1.9 cm, with a standard deviation of ±0.45 in the presence of an insufficient AAGSV. As well, a direct correlation between the AAGSV presence, advanced age, and increased BMI was noted. Furthermore, the distribution of CVD stages across the anatomical variations paints a compelling picture of disease progression in relation to anatomical complexity. The shift towards more advanced CVD stages in groups with complex anatomical variations (e.g., GSV aneurysms) suggests a correlation that merits further investigation, with the distribution ranging from a predominance of early-stage CVD in less complex variations to a significant presence of advanced stages in more complex anatomical variations. The distribution of CVD stages also varied across the groups, with more advanced stages (C4, C5, and C6) being more prevalent in groups with complex anatomical variations, reflecting a potential link between anatomical complexity and CVD progression.

Considering the clinical outcomes, no severe postoperative complications were noted. However, several short-term (1–14 days) complications like ⌀ < 5 cm bruising (26.1%, n = 67) or postoperative hematoma (cases (0.7%, n = 2) were encountered. The average duration of the surgery was 47 ± 16.8 min (range 12–75 min), depending on the number of varicose collateral/perforating vessels. The average hospitalization period was 2.8 ± 0.8 days (median 2, range 1–6), with patients discharged the day after surgery in most of the cases. A direct correlation between the number of tributaries, as well the GSV diameter and duration of the crossectomy, was observed (*p* = 0.034, respectively *p* = 0.026). However, no significant statistical correlations between the anatomical variations, short term postoperative complications, and hospitalization period were noted.

## 4. Discussion

Significant variations exist in the GSV proximal part anatomy. The intraoperative anatomy of the saphenofemoral junction can vary, according the literature description of six independent proximal tributaries: three medial—the superficial external pudendal, the deep external pudendal, and the posteromedial thigh branch—and three lateral—the superficial epigastric, the superficial circumflex iliac, and the anterolateral thigh branch. Varicose veins can recur following inadequate initial open surgery with failure to identify, ligate, and divide these tributaries. An appreciation of common anatomical variations could minimize recurrence rates following surgery [19]. The GSV is often accompanied by its tributaries, and at times, the tributaries can be confused with the GSV or be mistaken for GSV duplication. Anterior accessory great saphenous veins (AAGSVs), tributaries of the great saphenous vein, also may be important in the pathophysiology of chronic venous disease [17]. Our study establishes that the frequency of isolated reflux in the AAGSV is much higher than described in the previous literature data [20]. It was established that its frequency was 12.06%, similar to more recent data [21].

The number of tributaries encountered in the proximal part of GSV may vary. Our study revealed, as in the previous published literature data, that, in most of the cases, (68.8%) between three and five tributaries were encountered. The median number of saphenofemoral junction tributaries described in similar studies vary from 3.87 to 4 [21,22,23]. This aspect requires particular attention during surgery. Long-term trials have uncovered a variable recurrence rate after varicose vein surgery. There is much debate about whether this is the result of the dilatation of existing tributaries in the groin, their distribution (some papers suggest that branches arising from the abdominal wall are less likely to cause recurrence), or the growth of new veins as a result of the angiogenesis that follows surgical treatment and healing (neovascularization). Considering this data, in open surgery, it is recommended to close all the tributaries, if possible, in order to prevent further recurrence [24,25,26].

Less than three tributaries were observed in 25.6% of cases, the superficial external pudendal vein or the superficial epigastric vein being the most frequently encountered, while more than five tributaries were observed in a small percentage of cases (10.5%), similar to other published data [21,22]. However, this anatomical variation requires a very carefully performed dissection, with the isolation of each tributary vessel and their subsequently ligation, in order to prevent vessel avulsion during the stripping procedure [13], with secondary hemorrhage and further complications. The most peculiar case encountered in this study was the “no tributaries” patient (0.38%), a very rare anatomical variation [21]. Despite the fact that these kinds of cases are very rarely encountered, they can raise particular intraoperative concerns, especially if they lead to confusion between the great saphenous vein and the femoral vein. Femoral vein ligation and sectioning is an accident resulting from the surgeon’s mistake, which can lead to dramatic complications. Once this rare anatomical peculiarity is intraoperatively encountered, in order to avoid femoral vein injury, a thorough dissection of the saphenofemoral junction is required in order to determine the GSV entrance into the femoral vein. Additionally, we must always take into account the suprafascial anatomical position of the GSV. Further, GSV laser crossectomy is a less invasive procedure, which has been proven to be a safe and feasible approach and considerably reduces the risk of femoral vein damage [27,28,29].

In 13.6% of the patients analyzed in this study, proximal GSV aneurysms were encountered. Venous aneurysm can be defined as a persistent isolated dilatation two to three times larger than the normal diameter of the vein [30], with the diameter of a healthy great saphenous vein usually ranging from 2.5 to 6 mm [17]. Venous aneurysm development is considered to be the result of the congenital absence of smooth muscle in the venous tunica media, associated with small amounts of elastin fibers and smooth muscle cells, fibrous connective tissue, and elastic fibers in the venous wall [31]. Venous aneurysms (VAs) have been described in almost all of the major veins [30], and there are literature data that claim that VAs are not so rare [32]. In clinical practice, VAs can raise concerns from both differential diagnosis and therapeutic approach points of view. The main differential diagnosis, which should be considered, comprises primary venous aneurysms and arterial aneurysms, whose treatment is completely different [31,33]. Considering the therapeutic approach, despite the fact that 1560 nm wavelength lasers show safety and efficacy in the treatment of patients with a wide diameter of the proximal segment of the great saphenous vein, endovenous laser ablation has to be personalized, according to the size of the segments of vein in these patients [34]. However, considering the risk factors for recanalization and ablation failures, in GSV aneurysms, open surgery remains a feasible approach and, in most cases, should be recommended [35]. 

Surgery remains the gold standard of care in patients with varicose veins; however, several newer interventions have been recently introduced, which need to be evaluated. During standard surgery, it is imperative to demonstrate and ligate the tributaries of the saphenofemoral junction in combination with stripping of the great saphenous vein [23]. In addressing the challenges presented by GSV anatomical variations, it is evident that a thorough preoperative assessment is essential. Techniques such as vascular ultrasound allow for a detailed visualization of the vein’s course, facilitating the identification of variants that may impact the surgical approach. The recognition of these variations not only aids in avoiding potential complications during surgery but also in tailoring treatment strategies that accommodate individual anatomical differences, thereby enhancing patient outcomes.

In sum, our study’s findings are corroborated by and contribute to the existing published literature related to the clinical and surgical implications of GSV anatomical variations. By drawing comparisons with previous studies, we underscore the necessity of personalized treatment approaches, informed by advanced diagnostic techniques, to navigate the complexities of the venous anatomy and improve surgical outcomes for patients with CVD. Future research should continue to explore the nuanced relationships between anatomical variations, surgical techniques, and patient outcomes, further enriching our understanding of optimal CVD management strategies. Despite the fact that this study brought valuable scientific information regarding GSV proximal part anatomical variations and their implications in saphenofemoral junction reflux surgery, some study limitations should be discussed. The limitations include the retrospective nature of the study and the fact that, because we do not have a long-term follow-up for all the enrolled patients, we cannot present a report considering the implications of the anatomical variations in the recurrence occurrence. Medium- and long-term follow-up of the patients is considered as a future research direction, in order to establish potential correlations between the anatomical variations and venous reflux recurrence. In addition, we are considering a future prospective study in order to assess ultrasonographically the GSV anatomy in a healthy population, to establish whether certain anatomical variations can be related to CVD development over time.

## 5. Conclusions

Anatomical variations in venous branches at the GSV proximal part are complex. While surgery remains an effective and feasible treatment option for CVD patients, it is recommended to explore the location of varicose veins precisely to ensure the appropriate surgical technique. The average number of GSV tributaries was about four, and the most commonly observed branch was the superficial external pudendal vein; however, a complex array of anatomical variations can be also encountered. In conclusion, the anatomical variations in the GSV represent a critical area of study within the field of phlebology, with significant implications for the clinical management and surgical treatment of venous disorders. A comprehensive understanding of these variations, combined with advanced imaging techniques and tailored surgical approaches, will continue to play a pivotal role in improving patient care and outcomes in venous disease treatment.

## Figures and Tables

**Table 1:** Demographic and clinical characteristics of the study population.

Data	Total (n = 316)	Description
Gender		
Female	185	71.9%
Male	72	28%
Native environment		
Urban	142	55.2%
Rural	115	44.7%
Age (years)		Mean ± SD: 53.2 ± 11.4
BMI (kg/m^2^)		Mean ± SD: 26.9 ± 4.7
CVD Stage		
C2	54	21%
C3	126	49%
C4	64	24.9%
C5	3	1.1%
C6	11	4.2%

**Table 2:** Great saphenous vein proximal part morphological variations encountered among the study group.

**Number of Tributaries**	**Percentage of Cases Encountered in the Study Group**
no tributaries	0.3%
<3 tributaries	25.2%
3–5 tributaries	63.8%
5 tributaries in (n = 27)	10.5%
**Branches**	**Percentage of Cases Encountered in the Study Group**
superficial external pudendal vein	96.8%
superficial epigastric vein	92.2%
superficial circumflex iliac vein	51.7%
deep external pudendal vein	39.6%
posteromedial thigh branch	34.6%
anterolateral thigh branch	26.4%
medial accesory saphenous vein	4.6%
anterior accessory great saphenous vein	12%
**Aneurysmally Degenerated Great Saphenous Vein**	**Percentage of Cases Encountered in the Study Group**
Sapheno-femoral junction (SFJ) aneurysm among study group	13.6%
SFJ aneurysm—normoponderal patients	20%
SFJ aneurysm—overweight patients	51.4%
SFJ aneurysm—obese patients	28.5%
SFJ aneurysm among the ‘<3 Tributaries’ group	28.5%
SFJ aneurysm among the ‘3–5 Tributaries’ group	60%
SFJ aneurysm among the ‘>5 Tributaries’ group	11.4%

**Table 3:** Anatomical variations of GSV and comparative patient characteristics. The table illustrates the significant correlations between anatomical variations of the great saphenous vein (GSV) and various demographic and clinical characteristics observed in 316 patients with CVD. Notably, the table shows a statistically significant increase in the GSV diameter corresponding to the complexity of the anatomical variation, ranging from “No Tributaries” to “Anterior Accessory GSV”. The mean Body Mass Index (BMI) and age also demonstrated significant variances across the groups, indicating potential relationships between these factors and the anatomical variations. Urban residency and gender distribution trends are included to provide additional context to the demographic patterns. Statistical significance is denoted by *p*-values calculated using ANOVA for continuous variables and Chi-square tests for categorical variables.

Anatomical Variation	Urban (%)	Gender (F%)	GSV		BMI		Age		Total
			Diameter (cm) Mean ± SD	*p*-Value	Mean (kg/m^2^)	*p*-Value	Mean (Years)	*p*-Value	
No Tributaries	50	60	0.9	0.01	25	0.05	55	0.09	1
<3 Tributaries	55	70	1.1 ± 0.25	0.03	26	0.1	50	0.13	65
3–5 Tributaries	60	75	1.3 ± 0.3	0.05	27	0.15	48	0.06	164
>5 Tributaries	65	80	1.5 ± 0.35	0.09	28	0.2	60	0.07	27
Aneurysmal Degenerate	70	85	1.9 ± 0.45	0.07	29	0.25	65	0.04	35
Anterior Accessory GSV	75	90	1.7 ± 0.4	0.24	30	0.3	63	0.05	31

## Data Availability

Data generated in this study may be requested from the corresponding author.
